# Sensor-Based Assessment of Social Isolation and Loneliness in Older Adults: A Survey

**DOI:** 10.3390/s22249944

**Published:** 2022-12-16

**Authors:** Deepa Prabhu, Mahnoosh Kholghi, Moid Sandhu, Wei Lu, Katie Packer, Liesel Higgins, David Silvera-Tawil

**Affiliations:** Australian eHealth Research Centre, Commonwealth Scientific and Industrial Research Organisation (CSIRO), Brisbane, QLD 4029, Australia

**Keywords:** social isolation, loneliness, sensors, assessment, passive monitoring, activity markers, older adults

## Abstract

Social isolation (SI) and loneliness are ‘invisible enemies’. They affect older people’s health and quality of life and have significant impact on aged care resources. While in-person screening tools for SI and loneliness exist, staff shortages and psycho-social challenges fed by stereotypes are significant barriers to their implementation in routine care. Autonomous sensor-based approaches can be used to overcome these challenges by enabling unobtrusive and privacy-preserving assessments of SI and loneliness. This paper presents a comprehensive overview of sensor-based tools to assess social isolation and loneliness through a structured critical review of the relevant literature. The aim of this survey is to identify, categorise, and synthesise studies in which sensing technologies have been used to measure activity and behavioural markers of SI and loneliness in older adults. This survey identified a number of feasibility studies using ambient sensors for measuring SI and loneliness activity markers. Time spent out of home and time spent in different parts of the home were found to show strong associations with SI and loneliness scores derived from standard instruments. This survey found a lack of long-term, in-depth studies in this area with older populations. Specifically, research gaps on the use of wearable and smart phone sensors in this population were identified, including the need for co-design that is important for effective adoption and practical implementation of sensor-based SI and loneliness assessment in older adults.

## 1. Introduction

Social interactions and engagement are vital for human existence. The lack of human contacts or social connection is termed ‘social isolation’ (SI) [1]. SI refers to the disconnection or dissociation of an individual with their family, or members of the community, due to low levels of interaction or engagement. It is therefore considered as an objective and quantifiable concept that can be estimated by measuring network size, diversity, and frequency of interpersonal contact [2]. Loneliness, on the other hand, refers to the emotional state of feeling alone [3]. Perlman and Peplau [3] defined loneliness as ‘an unpleasant emotional state arising as a result of the qualitative and quantitative difference between the existing relations and the desired relations of a person with other people’. Loneliness is typically associated with a lack of sense of belonging, satisfaction, quality, and fulfilment in relationships. Therefore, loneliness is considered a qualitative and subjective concept that can be measured by quantifying the extent of meaningful relationships and the level of satisfaction in those relationships. SI and loneliness are two distinct but interrelated concepts, in that a person may have regular interactions with others and yet feel lonely due to lack of quality in the interactions. Being isolated from social networks for extended periods of time can lead to loneliness. Similarly, feeling lonely can cause people to detach from their social networks and become socially isolated.

Current understanding in the area of SI and loneliness highlights: (i) how older adults are disproportionately affected by these conditions; (ii) their serious implications on physical and mental health; and (iii) the need for timely identification of people at risk and reinforcement of support and interventions to prevent potential adverse outcomes. With an increasing ageing population and short-staffed aged care workforce, there is an increasing interest in leveraging the potential of modern sensors and technologies to autonomously support SI and loneliness assessment in older adults. With the aim of informing the development of effective methods for sensor-based assessment of SI and loneliness in older adults, this paper presents a survey of existing studies in this area. This survey covers the rationale of how sensor-based assessment of SI and loneliness has been achieved through measuring different behavioural and activity markers, the type of sensors used to detect them, the type of studies that have been conducted (including their duration, sample size, population, and important findings), and the gaps and limitations in the existing work.

### 1.1. Prevalence and Health Risks of SI and Loneliness in Older Adults

Social isolation and loneliness are recognised as one of aged care’s biggest challenges and are known to affect more than 20% of older Australians [4]. Projections show that SI is expected to affect half a million older Australians by 2040 [5]. According to the Australian loneliness and age report, the rate of loneliness increases by 40% in people aged 65 and older compared with younger populations, with prevalence progressively increasing with increasing age [6,7,8,9,10].

SI and loneliness are identified to be a risk factor for poor physical, mental, and emotional well-being [11]. SI and loneliness are strongly linked to increased risk of multiple health problems such as high blood pressure, cardiovascular disease, obesity, falls, a weakened immune system, anxiety, depression, cognitive decline, dementia, Alzheimer’s disease, and death [12]. Loneliness is also known to elevate mortality rates, comparable to other well-known mortality risk factors such as obesity [11]. SI and loneliness have implications far beyond the need for social support, particularly for older adults. Older adults are identified as one of the high-risk groups for SI and loneliness. Retirement, children living in different places, and loss of a spouse are identified as significant contributors to the SI and loneliness risks in older adults. Other contributors include physical and cognitive decline, sensory impairment, chronic illness, urinary incontinence, and insomnia. These factors are also known to reduce opportunities for older adults to engage socially (see [13] for a review on factors contributing to SI and loneliness in older adults). Older adults are at an increased risk of serious health complications when exposed to communicable diseases, such as COVID-19. Hence, in the interest of protecting their health, older adults were imposed with high levels of restrictive physical distancing measures and in-home confinement during the COVID-19 pandemic lockdowns. This has exacerbated the health, as well as SI and loneliness, risks in older adults due to restricted movements, reduced physical activity, and decreased social interactions.

### 1.2. Impact of SI and Loneliness on Aged and Health Care

SI and loneliness increase the demand for aged and health care resources that are already facing severe shortages. The management of SI and loneliness incurs both personal and societal costs [14], including management of chronic, acute, and debilitating diseases [12], the increased use of emergency services [15,16], and the risk of admission to residential aged care [17]. Appropriate monitoring and timely management of SI and loneliness have the potential to reduce aggravated and complex health impacts that could prevent unnecessary hospital admissions [15], reduce general practitioner (GP) visits [16], and lower the likelihood of older people requiring entry into to residential aged care [17].

### 1.3. Existing Assessment Approaches for SI and Loneliness and Their Limitations

Many validated questionnaire-based self-report assessments of SI and loneliness exist, such as the University of California Los Angeles (UCLA) Loneliness Scale [18], De Jong Gierveld Scale [19], and Lubben Social Network Scale [20]. These scales rely on one-on-one in-person interactions, which limits their applicability in routine practice. In fact, none of the available questionnaires are used in mainstream care as part of routine assessment in Australia and globally [21] due to: (i) an already over-stretched short-staffed aged care workforce, (ii) the stigma associated with being identified as someone experiencing SI or loneliness [12], (iii) people’s discomfort in sharing their vulnerabilities during a face-to-face interaction [22,23], and (iv) the increased risk of exposure to communicable diseases (e.g., COVID-19) [6]. Additionally, there are concerns around the accuracy and reliability of subjective responses to questionnaires and the influence of social-desirability bias, memory problems, under- or overestimation, cognitive status, disease status, and mobility challenges [24,25]. In the current system, clinicians, caregivers, family members, or service providers typically initiate assessment and support on an ad hoc basis when they pick up on some of the signs during regular interactions with the individual. There is also a lack of a systematic and objective approach to monitor and evaluate the outcomes of people during and after receiving intervention.

### 1.4. Sensor-Based Assessment of SI and Loneliness

Sensor-based assessment of SI and loneliness refers to techniques used to identify people experiencing SI and loneliness based on changes in activity and behaviour patterns caused by SI and loneliness through different types of sensors (including ambient and wearable sensors). In recent times, there has been an increasing interest in employing sensor-based methods for assessing SI and loneliness in older adults [26,27]. These methods allow identification of people at risk, monitoring of the impact and outcomes of interventions, measuring their outcomes, and generating individual-, group-, and population-level data informing the prevalence and care delivery for SI and loneliness. Sensor-based approaches have the potential to facilitate unobtrusive screening of SI and loneliness in a dignity- and privacy-preserving manner with reduced human intervention and less dependence on human resources.

To date, there is limited work in this area, particularly with older adults. Currently, sensor-based assessments in the older population have mostly focused on using sensors to monitor activity patterns. There have been some studies in younger adults using sensors to assess SI and loneliness, however, there is limited knowledge of the specific activity and behavioural markers that could be used to assess social isolation in older adults. While ambient and wearable sensors are of great interest in activity and behaviour monitoring, the feasibility and accuracy of using these types of sensors have not been explored in-depth in older population. Additionally, the literature in this space lacks evidence from longitudinal studies with validated results from a large and diverse population. The aim of this paper is to examine previous studies in the area of sensor-based assessment of SI and loneliness in older adults and provide an overview of the activity and behavioural markers of SI and loneliness, the types of sensors, study design, study duration, sample size, and their findings. The results of this survey inform the existing evidence, feasibility of the methods, limitations, gaps, and opportunities for future work.

### 1.5. Comparison with Existing Surveys

The present survey differs from recently published surveys by Qirtas et al. [28] and Site et al. [29]. The survey by Qirtas et al. [28] explored previous work in passive sensing techniques to detect SI and loneliness in younger adults, older adults, and mixed age groups, whereas our survey specifically focuses on older populations. This difference by itself sets our paper apart in the type of markers, sensors, and study designs that are specifically applicable to older population. The paper by Qirtas et al. [28] did not review the duration of the studies, sample size, data analysis methods, and co-design components of the existing studies; instead, the authors focused on the types of sensor-based assessments used for SI in all age cohorts. Finally, the recent survey by Site et al. [29] is broader in scope and includes monitoring and management of SI using wearable solutions. Their survey, while being specifically focused on wearables and social isolation, had a multidisciplinary perspective involving technology, gerontology, socio-psychology, and built environments. Their paper also has a specific focus on applying machine learning (ML) models to wearable sensor data collected with the aim of monitoring and managing SI. In comparison, the current survey focuses specifically on sensor-based assessment of both SI and loneliness in older adults and explores the markers, sensors, study design aspects, and validity of the results that can be applied to develop sensor-based screening tools.

## 2. Methods

### 2.1. Search Strategy, Data Sources, and Screening

**Figure 1:**
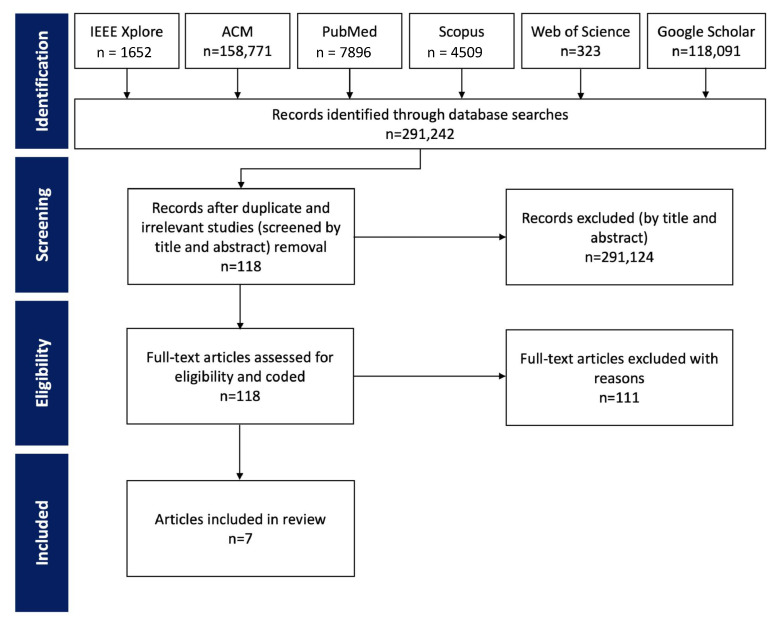
PRISMA flow diagram of the article identification and selection process.

To conduct this survey, we followed the PRISMA [30] and PRISMA-ScR [31] guidelines. The PRISMA flow diagram illustrating the selection process is shown in Figure 1. The databases IEEE Xplore, Scopus, ACM, PubMed, Web of Science, and Google Scholar were searched for the initial identification of relevant publications. The search query applied was as follows:

- Social isolation AND (assess* OR monitor* OR detect* OR identi* OR sens*);
- Loneliness AND (assess* OR monitor* OR detect* OR identi* OR sens*).

Articles published between January 2010 and October 2022 were extracted and screened by their title to remove duplication and non-relevant studies by two authors. The remaining articles were then coded by two different authors to identify articles in consented categories of behavioural markers of social isolation, sensor-based assessment of SI and loneliness, intervention, machine learning (ML) modelling, and other approaches to identify and predict SI and loneliness.

### 2.2. Inclusion and Exclusion Criteria

Only studies published in English with a focus on SI and loneliness assessment through sensing technologies (such as smartphone applications, ambient sensors, wearables, etc.) were included in this survey. Studies were excluded if their focus was on interventions for SI or non-sensing approaches for SI and loneliness assessment. This survey only included papers containing social isolation or loneliness terms and excluded papers that measured different constructs of the concepts of SI and loneliness.

### 2.3. Data Extraction

As shown in Figure 1, a total of 291,242 articles were identified using the search queries. After removing duplicates and screening their title and abstract, 118 full texts were reviewed and coded to differentiate intervention-based studies, assessment/prediction-based studies, and those with the focus on behavioural marker of SI and loneliness. Finally, 7 articles with the focus on sensing-based technologies for SI and loneliness assessment were selected for inclusion in this survey.

## 3. Results

### 3.1. Rationale Applied in Sensor-Based Assessment of SI and Loneliness

Extensive evidence shows that SI and loneliness are characterised by altered lifestyle behaviours and activity patterns such as physical inactivity, altered sleep patterns, reduced conversational behaviour, low mobility and walking speed, less time spent away from home and in different parts of the home, and changes in eating and cooking behaviours [7,8,9]. A list of behavioural and activity markers that have been shown to be correlated with SI and loneliness is given in Table 1.

Based on this evidence, previous studies deployed different types of sensors including wearable fitness trackers, smart watches, smartphones, and ambient devices equipped with accelerometers, gyroscopes, temperature, and humidity sensors that can generate objective data to facilitate pervasive measurements of behaviour and activity markers of SI and loneliness. Such devices have become ubiquitous in everyday life in recent times and are being extensively used to track activity patterns including sleep, proximity between people, and physiological information (such as heart rate, respiration rate, temperature, and oxygen saturation) [41,42,43]. The studies included in this survey used statistical or mathematical techniques to infer if a person is experiencing SI or loneliness by comparing the collected behavioural and activity data from sensors with results acquired from standard scales.

### 3.2. Categories of Measured Activity Patterns

Sensor-based assessment of SI and loneliness predominantly rely on estimating SI and loneliness through activity and behaviour patterns using sensors. Based on the type of activity used for estimating SI and loneliness, previous work investigated the following five categories of activities performed by older adults that were measured using sensors: (i) in-home activities, (ii) out-of-home activities, (iii) sleep patterns, (iv) phone usage, and (v) others. The first four categories (shown in Table 2) include activities that were measured using sensing devices. The fifth category (named “others”) is described in Section 3.2.5, which includes other automatic non-questionnaire-based methods to assess SI and loneliness, such as machine-learning- and computational-modelling-based approaches (using synthetic/existing data) and verbal behaviour and natural language processing approaches. Each of these categories are discussed in the following subsections.

#### 3.2.1. In-Home Activities

In-home activities are one of the most common types of activities measured in sensor-based assessment studies as an indicator of SI and loneliness. Five out of seven studies included in our survey [26,37,46,48,49,50] measured different activities performed by older adults inside a home setting through sensors. Some of these activities include the amount of mobility [44], walking speed [37,44], activity levels, and time spent in different parts of the home (such as living room, bedroom, kitchen, etc.) [26,27,45,46]. The rationale behind measuring these activity patterns is based on the evidence from SI and loneliness behavioural studies that suggests that people experiencing SI and loneliness tend to become physically inactive and exhibit sedentary behaviours [7,34], spend more time in bed and living rooms [51,52], and show less activity in the kitchen [36].

#### 3.2.2. Out-of-Home Activities

Time spent out of home was one of the most widely explored activities (by six out of seven studies [26,37,44,46,47]). Two studies looked at the frequency of going out [26,37], and one study looked at the time spent in garden [27]. It is worth mentioning here that all of the studies that measured out-of-home activities did so using ambient sensors installed in the older adults home (such as door contact sensors), and none of the studies used sensors (e.g., wearables) to specifically track movements outside of the home.

#### 3.2.3. Sleep Patterns

Studies on understanding behaviour changes in people experiencing SI and loneliness have reported a change in sleep patterns, such as increased day-time napping, reduced sleep duration during the night or increased movement during the night [7,35]. Along these lines, the study by Goonawardene et al. [26] has investigated measures such as time spent in bedroom and nocturnal movement using ambient sensors.

#### 3.2.4. Phone Usage

There is existing evidence showing phone usage patterns as an indicator of socialisation [39] and that this can be used to infer a user’s mood and mental health [53]. Studies included in this survey explored features such as the number and duration of incoming and outgoing calls [27,39,44], number of incoming and outgoing text messages [27], and persons from whom calls or texts were received or sent [27]. These features were used to estimate the amount of social interactions a person has over the phone and closeness to those people (e.g., whether the outgoing or incoming calls were from family, friends or others).

#### 3.2.5. Others

This survey identified a category of studies for automatic assessment of SI and loneliness that did not use sensors or sensing devices. These studies, however, developed predictive machine learning models that were based on either synthetic data, data from existing databases, or validated surveys (subjective data). While these studies are not included in this survey, they are mentioned here to inform readers about the other types of SI and loneliness assessment methods being developed. Zadeh et al. [54] built a computational model to identify SI in older adults using synthetic social network data. In another study, databases that include older adults’ online responses to SI and loneliness questionnaires were used to build predictive models using machine learning tools [55]. Similar machine-learning-based approaches were developed by Yang and Bath [56] to predict loneliness in older adults using existing data collected from a large longitudinal study. In addition to the predictive modelling, previous works have explored the identification of SI and loneliness in older adults through verbal behaviour analysis. A study by Badal et al. [57] explored the assessment of SI and loneliness in older adults using natural language processing of interview data [57]. Yamada et al. [58], on the other hand, developed a tablet-based application that collects audio data on speech responses to daily life questions, which was then analysed and correlated with self-rated scores of the UCLA Loneliness Scale [58].

### 3.3. Standard Scales Used to Measure SI and Loneliness

Questionnaire-based scales that can be used to self-report, or can be administered during an interview, are a standard way of assessing SI and loneliness. They have been used in sensor-based assessment studies to collect the ground truth on the level of SI and loneliness experienced in different population groups. This ground truth is then used to compare with the performance of the sensor-based assessment methods. Various validated questionnaire-based scales exist for this purpose, and a list of the commonly used scales is given in Table 3. The University of California Los Angeles (UCLA) Loneliness Scale and the De Jong Gierveld (dJG) Scale are the two most commonly used scales in the studies included in this survey, with the UCLA scale being used in four studies and the dJG scale used in two.

The UCLA Loneliness Scale measures subjective feelings of loneliness using a 20-item (10 negatively scored and 10 positively scored items) questionnaire. This scale is a revised version of the original scale [18] and is referred to as the R-UCLA scale. The positive and negative scoring refers to the wording of the questions, such as, ‘I feel isolated from others’ (negative scoring) and ‘I do not feel alone’ (positive scoring). The dJG scale is an 11-item questionnaire [19] that assesses two aspects (i.e., emotional and social) of loneliness. This scale is a revised version of the original 34-item scale [59]. Similar to UCLA, dJG consists of positively and negatively scored items. For example, ‘There is always someone I can talk to about my day-to-day problems’ (positively scored item) and ‘I experience a general sense of emptiness’ (negatively scored item). The other scale that has been used for measuring loneliness is the ESTE-R scale [60].

**Table 3:** Standard instruments used to measure social isolation and loneliness.

Standard Scales	Parameter Measured	Article
University of California Los Angeles (UCLA) Loneliness Scale	Loneliness	[44,46,47,48]
De Jong Gierveld Scale	Loneliness	[26,37]
ESTE-R scale	Loneliness	[61]
Lubben Social Network Scale	Presence of social network	[26,27]
Custom survey	Frequency of attendance in social events	[26]
**Other Scales**		
Single-item measure of social identification	One’s positive emotional valuation of the relationship between self and in-group	[62]
Friendship scale	Perceived social support from family and friends	[63]
The social support questionnaire	Social support satisfaction, social participation and material aid	[64]
Inventory of Socially Supportive Behaviours	Instrumental, informational, and social support	[65]

One of the concerns reported in the use of loneliness scales is their validity across different populations and age groups [66]. Since social connection patterns and perceptions of loneliness differ in various age and ethnic groups, the validity and use of the same scales across all different ethnic and age groups has been questioned. Penning et al. [66] compared the measurement of loneliness in middle-aged and older adults using UCLA and dJG scales in their study and reported that ‘*most methodological work conducted using the R-UCLA scale has drawn on younger adult samples (Hawkley et al. [38] is an exception), much of that conducted using the dJG scale is based on older adult samples, particularly from Europe*’. A systematic study confirmed the validity of UCLA in older adults population [67].

SI scales include the Lubben Social Network Scale [20], the single-item measure of social identification [62], the friendship scale [63], and the social support questionnaire [64]. The Lubben Social Network Scale is an instrument used to measure the presence of social networks and engagement in social activities and is extensively validated across different population groups and in older adults [68,69,70]. A 12-item and 6-item version of the scale exist, and it is one of the most widely applied tools used to measure SI. Two of the studies [26,27] included in this survey that investigated SI used the Lubben Social Network Scale to investigate SI through social connectedness. The Lubben Social Network Scale is extensively validated across different population groups and in older adults [68,69,70].

### 3.4. Study Designs

This survey identified seven sensor-based studies to assess SI and loneliness in older adults. The study type, method, population group, sample size, study duration, and co-design aspects employed in these studies were extracted for further analysis. A summary of the study design aspects is presented in Table 4. Most of these studies conducted feasibility trials to demonstrate the applicability and reliability of sensor-based approaches in objectively measuring the behavioural and activity markers of SI and loneliness. They mostly employed a quantitative approach to examine association of the measures inferred from sensor data against subjective measures of SI and loneliness extracted from standard survey instruments and questionnaires.

Walsh et al. [45], for example, conducted a home-based feasibility trial of a smart home configuration installed in 13 older adults’ (aged over 60 years old) homes for 28 days to explore which measures of activities of daily living correlated with older adults’ outcomes, such as loneliness. Similarly, Austin et al. [44] conducted long-term trials in order to build a proof-of-concept system to estimate loneliness. In this work, they collected longitudinal data using in-home sensors from 16 older adults (aged 62 and above) for a period of 8 months. Furthermore, Goonawardene et al. [26] and Huynh et al. [37] deployed an unobtrusive sensor-based system (low-cost and privacy-preserving, with minimum involvement of the older adults) in 50 homes of independently living individuals aged 65 or above living independently for a period of 6 months. During this period, they collected the participants’ in-home mobility patterns. They employed a quantitative approach to correlate subjective and objective data collected from surveys and ambient sensors, respectively, to detect SI and loneliness in older adults. Goonawardene et al. [26] adopted a mixed-method approach in which they additionally validated the results of their quantitative approach with qualitative data collected from home visits.

Only three studies were found in which predictive models of SI and loneliness were built and evaluated. Petersen et al. [46] first developed a predictive model of loneliness by installing motion-activated video cameras and contact and motion sensors in the home of four older adults for 30 days to collect ground truth. Then, they evaluated their model by collecting subjective and objective data from 34 older adults over a period of five days. Martinez et al. [27] also conducted a small-scale feasibility trial of their predictive model for automatic detection of SI in seven older adults (aged between 60 and 74 years old) for one week. They monitored and detected unusual behavioural and daily activity patterns in older adults through a mobile application. In another study, Sanchez et al. [61] developed predictive models to infer the level of loneliness in older adults through activities captured by a smartphone. They developed a mobile application to collect data about four main factors of loneliness related to family, spousal, social, and existential crisis. They conducted a small-scale feasibility trial of their application and predictive models with 12 older adults (aged between 60 and 89 years old). In this trial, participants were asked to install the mobile application on their smartphone and use it for one week during their daily routine.

### 3.5. Data Collection Approaches

Different types of sensors, including motion and contact sensors, were used to monitor behavioural and activity patterns of older adults across the seven studies included in this survey. A summary of the different activity patterns measured through sensors is provided in Table 5, and the types of sensors and their installation locations are given in Table 6. Most of the studies deployed motion sensors in the rooms of a home and contact sensors on the main entrance [26,37], on external door(s) of the home [44], and on the internal doors of living room, main bedroom, and en suite [45,46]. These sensors captured information related to patterns of sleep, toileting, activities in the kitchen, time spent in the living room, and going out. Some of the other sensors included light switch and electricity (current flow) sensors. Walsh et al. [45] installed light switch sensors and water and electricity flow sensors installed on water faucets, as well as in the living room, kitchen, hall, bedrooms, and en suite. Austin et al. [44] also deployed phone monitoring devices in each home to unobtrusively assess daily phone usage, as well as a computer software to monitor all computer-related activities.

Using these sensors, researchers were able to extract a range of different features (Table 5). Goonawardene et al. [26] extracted the time spent in the living room, activity level in the kitchen, sleep duration (daytime napping and nighttime sleep), going out duration (average daily away time), and away count (the number of times participants went out), whereas Huynh et al. [37] focused on the the ratio of time spent inside and outside the home. Huynh et al. [37] only collected data through the Geriatric Depression Scale (GDS) [71] and De Jong Gierveld (dJG) Loneliness scale, while Goonawardene et al. [26] collected demographic and other information related to older adults’ physical and mental well-being through a survey. Additionally, Goonawardene et al. [26] measured SI as a composite measure of (i) relative lack of a social network, including both family and friends, (ii) subjective loneliness, and (iii) absence of social activities. All three aspects were assessed using the following self-reported measures: (i) the Lubben Social Network Scale, (ii) the dJG Loneliness Scale, and (iii) a survey on older adult’s frequency of attendance in four different activities of meeting friends, visiting family, attending religious activities, and having meals outside. To complement their assessment, they collected data through the GDS to measure geriatric depression (found to be associated with loneliness), the Abbreviated Mental Test score to assess cognition (a possible outcome of perceived social isolation), and other well-being parameters including subjective sleep quality, chronic conditions, activities of daily living (ADL), and instrumental activities of daily living (IADL). Additionally, Goonawardene et al. [26] collected the ground truth of the residents’ daily routines through two home visits per month.

Austin et al. [44] extracted different features from sensors, including daily hours spent outside the home, number of incoming and outgoing phone calls, in-home walking speed, in-home mobility, time spent on computer, and number of sessions on computer. They also collected data from UCLA loneliness scale at four distinct time points during the longitudinal trial. Walsh et al. [45] collected information related to participants wellbeing through a range of questionnaires, including the Hospital Anxiety and Depression Scale, the Pittsburgh Sleep Quality Index (PSQI), the Centre for Epidemiological Studies Depression Scale, the De Jong Giervald for loneliness, the Montreal Cognitive Assessment, the Short-Form Survey (SF-36) for quality of life, and the Katz Index of Independence in Activities of Daily Living. They extracted the following features from ambient sensors: (i) percentage of time spent in each room/location, (ii) number of transitions between locations, (iii) total duration of activity, and (iv) total duration of nocturnal activity. Petersen et al. [46] assessed the level of loneliness in their study using the UCLA loneliness scale. They also extracted entry and exit events from sensors and calculated the time out of home to develop their model.

In two other studies, mobile applications have been developed to monitor older adults’ behaviours. Martinez et al. [27] developed a smartphone application with four modules: (i) registration, in which the demographic data are collected and older adults add the phone numbers of family members and friends that they used to contact using their smartphone; (ii) message retrieval, in which variables such as outgoing messages sent to friends and incoming messages received from family are extracted; (iii) call pickup, in which variables such as average number of incoming calls from family, average duration of incoming calls from family, average daily number of incoming calls from friends, and average duration of outgoing calls to family are extracted; and (iv) home monitoring, in which variable including average time in the bedroom, average time in the living room, average time in the dining room, average time in the garden, and average time in other areas of the home are obtained using Bluetooth devices in an automatic manner. The sensors were installed in the following rooms: bedroom, living room, dining room, garden, and other home areas such as the studio, garage, kitchen, and cellar. In these studies, the Lubben Social Network Scale was used to collect information on the level of social isolation in participants. In a different study, Sanchez et al. [61] developed a mobile application to collect information related to phone calls, which included type of calls (incoming or outgoing) and the telephone number in order to identify it as a family member, friend, or acquaintance. It also collected the geographical location of the older adult using the GPS sensor in their smartphone.

### 3.6. Data Analysis and Findings

Most studies explored associations of the data collected through sensors and/or mobile applications with subjective data collected through standard instruments [46]. The data analysis methods used and the key findings of each study included in this survey are summarised in Table 7. Petersen et al. [46] investigated the correlation between time spent outside the home collected through sensors and loneliness derived from the UCLA Loneliness Scale through logistic regression and correlation analysis. They found that average time outside the home is negatively correlated with the loneliness score. Similarly, Walsh et al. [45] conducted correlation analysis and Principle Component Analysis between each feature derived from sensor data and data derived from the standard scale. They found that the increased time spent in the living room and nocturnal movement were associated with increased loneliness. Huynh et al. [37] conducted a correlation analysis that showed ‘room-level movements within a house’ and ‘going out’ behaviour captured by sensors can potentially detect severe cases of loneliness and depression.

Furthermore, Austin et al. [44] built linear mixed-effects regression models using the longitudinal in-home sensor data and the UCLA Loneliness Scale. They found that time spent outside the home is negatively correlated with loneliness. Longitudinal analysis of data showed that daily time out of home and number of computer sessions were significantly associated with loneliness. However, no association was found between phone/computer usage and loneliness. Goonawardene et al. [26] employed a mixed-method approach by conducting a two-step data analysis: (i) a quantitative analysis to investigate association between the sensor-derived features and subjective measures of wellness and social isolation collected through questionnaires, and (ii) an in-depth qualitative analysis to validate the findings from the quantitative analysis. A personalised profile per participant was generated based on the data from periodic observations, interviews, and surveys to enable the qualitative analysis. They found that the average time spent outside home is associated with social loneliness and the social networking score of older adults. Moreover, average time spent in the living room was found to be significantly associated with the perceived emotional loneliness level amongst older adults. These findings were further validated by analysing sensor data qualitatively through observations and through analysing survey and interview data related to in-home daily living patterns.

Sanchez et al. [61] and Martinez et al. [27] built predictive models and evaluated their performance against SI and loneliness scores from standard instruments. Martinez et al.’s [27] predictive model achieved 100% precision in detecting all levels of SI captured by their mobile application and it compared against the Lubben Social Network Scale as the ground truth. However, the sample size was very small. Sanchez et al. [61] evaluated the performance of their predictive models against the standard reference (the ESTE-R scale). They found that average time spent outside of the home and total of outgoings are the most important attributes for inferring loneliness. Outgoing and incoming family calls were found to be relevant attributes for family and spousal loneliness. For social loneliness, acquaintances calls were identified as a relevant attribute, whereas friends’ calls were found to be a non-relevant attribute.

## 4. Discussion

This survey identified a total of seven empirical studies reported in the period between January 2010 and October 2022 that met the inclusion criteria. This suggests that the area of sensor-based assessment of SI and loneliness in older adults is fairly under-explored. Two out of the seven short-listed studies focused on the assessment of SI, and five focused on loneliness. While both (sensor-based assessments of SI and loneliness) are really small in number, sensor-based assessment of loneliness in older adults was relatively more explored in comparison with sensor-based assessment of SI.

### 4.1. Sensor-Based Activity Features

This survey identified some of the sensor-based activity features of interest that applied to the assessment of SI and loneliness. The most commonly used features were time spent out of home (6 out of 7 studies) and time-spent in different parts of the home (5 out of 7 studies). These studies found a strong association of time spent out of home with loneliness [37,44,46] and SI [26]. However, the use of going-out behaviour as an indicator of SI and loneliness needs to be re-evaluated in the context of situations such as COVID-19 lockdowns, where people spent extended periods of time at home with reduced opportunities to venture out of home and interact with people. Time spent out of home is usually measured through motion and door contact sensors. However, these sensors work best in a single-occupancy scenario and could potentially give rise to spurious recordings in multioccupancy situations [72,73]. Other approaches to calculate time spent away from home using tools such as wearable location trackers remain unexplored. Emotional loneliness was found to be associated with in-home activity features such as time spent in different parts of the home [26], poor sleep quality or nocturnal activity [26,45]. A few studies explored computer or phone usges that were found to be associated with loneliness [27,44]. Social media usage is a virtual form of social engagement and has been shown to negatively affect loneliness [74]. While studies included in this survey have explored computer usage and phone usage, social media usage through these devices has not been measured and explored. It is worth mentioning that all of the seven studies included in this survey were conducted with older adults living independently. Therefore, the following results are derived from, and are representative of, activity patterns of independently living older adults. In comparison with the independently living, the activity of older adults living in different settings such as retirement villages or residential-aged care patterns could be different.

### 4.2. Effect of Confounding Variables

This survey focused on measuring SI and loneliness through changes in behaviours and activities. Mobility [26,37,44,45,46], physical activity [26,45], sleep [26,45], and phone usage [44] were some of the primary activity patterns that were measured to assess SI and loneliness through sensors. However, it needs to be acknowledged that changes in activity patterns may have various different causes in older adults. Hence it may be challenging to establish if a change in these activity markers is due to SI and loneliness or whether there are other contributing factors, such as a disease process or increasing frailty. As a result, when using such activity patterns and behaviours as a reference in older population, different causes of a change in behaviour need to be considered. For example, decreased time out of home and time spent in different parts of the house could be due to reduced mobility, secondary to increasing frailty, and not due to SI and loneliness. The same issue applies to behaviours such as sleep and phone usage.

The influence of such confounding factors on accurate estimation of SI and loneliness through sensor-based assessments has been acknowledged by Goonawardene et al. [26] and Petersen et al. [46] in their respective studies. However, none of the studies included in this survey account for the effect of confounding variables, such as disease and health status, that might result in similar behavioural patterns in participants to those seen in loneliness and SI. Accounting for confounding variables by establishing a baseline status of activity levels and behaviours that are relevant to sensor-based assessment of SI and loneliness could increase the accuracy of estimation. Additionally, validating, testing, and refining the sensor-based assessment models through longitudinal, diverse, and large cohort studies could help to identify other demographic features that are likely to influence the accuracy of SI and loneliness detection.

### 4.3. Sensor Measurement Accuracy

Sensor-based methods for assessment of SI and loneliness rely on accurate sensor measurements for reliable and consistent estimation. However, activity and behaviour measurements through sensors suffer from measurement inaccuracy. In studies using ambient sensors, inability to accurately track and localise the activity patterns of persons of interest in multioccupancy scenarios has been reported. For example, Huynh et al. [37], Walsh et al. [45], and Austin et al. [44] reported that detection of movement and activity patterns using PIR and door contact sensors worked accurately only in single-occupancy environments. The presence of visitors, multiple occupants, or pets led to recording multiple activity events from the sensors that were not related to the subject of interest. Due to the limited research in sensor-based assessment of SI and loneliness in older adults, the problem of acquiring accurate location, identification, and tracking of subjects of interest has not been well-explored. In this vein, research in the area of activity recognition in smart homes [72,73,75,76] has widely reported and investigated the challenges in accurately identifying and tracking people in a multioccupancy sensor-based environment. Examples include vision-based (i.e., identifying people performing activities through biometric features (e.g., face) derived from image or video information from cameras), signature-based (i.e., through unique activity patterns specific to a subject (e.g., gait) recorded through different types of wearable sensors), and tag-based approaches (i.e., wearable wireless tags that are detected and recognised across receivers placed in different parts of the house through a unique ID) [77]. While privacy and accuracy concerns are reported for the vision-based and signature-based approaches, respectively, tag-based approaches are considered more promising [77]. Nevertheless, tag-based approaches require the person to wear and charge the device regularly, which could be challenging for older adults with cognition and memory impairments [44]. Future research on sensor-based assessment of SI and loneliness could benefit from adopting proven methods from smart home research to identify and track activities of older adults.

Studies associating phone usage behaviour with SI and loneliness aim to understand the level of social interactions occurring through phones. Hence, studies on phone usage behaviour need to accurately capture details, such as number and duration of calls made and received, people to whom calls were made and received from, number of texts sent and received, and people to whom texts were sent and received from. While this information may be possible to capture on a mobile phone, it might not be straightforward to capture information on whom the calls were made to and received from on a landline phone. Austin et al. [44] reported that the results of their study were affected by their limitation of capturing such details on mobile phone usage. With regard to landline phone usage, Petersen et al. [48] reported that it was possible to record the phone ring, the number of times a phone was taken off the hook (incoming call), and if a number was subsequently dialled (outgoing call). However, their study mentioned that it was difficult to establish if an outgoing call was answered. They further report that the phone monitoring software was limited by its capacity to record the dialled number and duration of the call. They also expressed concerns over using landline phone usage to measure SI and loneliness in cohorts that are more inclined towards using mobile phones as a preferred medium for communication. With regard to associating computer usage with SI and loneliness, Petersen et al. [48] reported that total time of computer usage should also take into consideration the time spent on social networking activities on smart phones and tablets.

### 4.4. Privacy

Privacy preservation is one of the important requirements of any technology-based application. The studies included in this survey have reported due diligence in acquiring consent before using sensors to record activities in participants’ homes. In the interest of privacy, it is also important to note that the studies included in this survey did not include video data. PIR and door contact sensors used in [26,37,44,45,46] are passive and non-intrusive in recording activity data. Some of the approaches used may raise privacy concerns or be considered too intrusive (e.g., collecting information about calls made and received or location-based information in [27,61]). Future work in sensor-based technologies needs to consider privacy-preserving mechanisms such as smart card, symmetric key encryption, and digital signature [78] in their study design to ensure the data security and integrity.

### 4.5. Study Design and Data Collection

The current work in the area of sensor-based assessment of SI and loneliness has been conducted with the independently living older adults and has not been explored in those living in residential aged care and retirement villages. While residential aged care facilities and retirement villages offer some level of social engagement opportunities (such as meal-time interactions and group activities), the problem of SI and loneliness in this population continues to exist and has been widely reported [79,80]. Additionally, people accessing residential care typically have higher care needs compared with those living independently in the community, and it is therefore likely that the activity and behaviour patterns that are indicative of SI and loneliness in this population are different to the patterns seen in independent living population. Another important observation of the studies reviewed in this survey is that they incorporate small sample sizes and short study durations. With the exception of Austin et al. [44], who conducted a long-duration study (between 6 to 8 months) with a sample size of 16 participants, all others were short-duration studies ranging between 5 to 28 days. The need for extensive studies with a diverse and larger sample size to achieve higher accuracy [27,44,48,61] and reduce over-fitting effect [61] has been reported. The outcome of studies with a small sample size may also be found to be difficult to replicate. Sanchez et al. [61] expressed concerns regarding sensor data collection with older adults as being time-consuming and expensive, which could be the reason behind short study durations and small sample sizes seen in the previous studies. However, it needs to be noted that smaller sample sizes and shorter study durations are limitations in this area at present. Large cohort longitudinal studies will allow the creation of a larger data set that can be used to build accurate SI and loneliness prediction tools and can also offer opportunities to apply machine learning techniques that are representative of a diverse population.

### 4.6. Complementary Self-Report Data

In the context of sensor-based assessments of SI and loneliness in older adults, limited focus on self-report data about daily personal and environmental factors is one of the observations made in this survey. With the exception of Walsh et al. [45], who collected self-reported questionnaires, the studies included in this survey did not collect such data (e.g., weather, visitors, and household composition) [81]. Self-report data are considered to be an important indicator of external context variables and potential confounding factors that could potentially influence the interpretation of an individual’s health and mental status. To draw a more accurate and personalised picture of all of the factors that have a role in each individual’s SI and loneliness assessment, it is essential to collect such self-report data in order to complement the sensor-based objective data.

### 4.7. Validity

Validation is an important step in the development of sensor-based assessment tools to assess SI and loneliness that can inform their implementation into practice. Validation helps to establish how well the results of the developed tools are applicable to diverse and larger population and compare with the ground truth data which are acquired using standard scales to assess SI and loneliness. All the studies included in this survey measured SI and loneliness through validated standard scales and used the derived scores to compare the validity of their sensor-based approaches. However, these studies are few in number and involve small sample sizes. Therefore, large cohort studies are needed to understand the effects of other demographic variables and health and disease status to establish the applicability of their results to a wider population.

### 4.8. Co-Design, User Acceptability, and Digital Literacy

Co-design and human-centred approaches amplify technology users’ voices in the design process by considering them as active participants/collaborators in the study. In the interest of increasing acceptance and use, research in co-design has explored how to engage older adults in the design process of technologies for ageing in place. The present survey explored considerations on user acceptability, co-design processes, and the influence of digital literacy in the existing body of work on sensor-based assessment of SI and loneliness. The study by Lyons et al. [47] included a brief questionnaire to collect qualitative data about user perceptions on acceptability and usability. However, this study did not extensively and systematically explore the usability of their proposed sensor-based approach for SI and loneliness assessment. Other studies did not report on usability. None of the studies included in this survey implemented co-design processes or considered the effect of digital literacy among older adults on the development and use of sensor-based assessment of SI and loneliness.

### 4.9. Nuanced Concepts of Loneliness—Gaps and Challenges in Sensor-Based Measurements

In this Section 1, we provided an introduction to the concepts of SI and loneliness. While these conceptual definitions are widely applied in the sensor-based assessment studies reviewed in this paper, the existence of more nuanced concepts such as emotional loneliness, social loneliness, and existential loneliness needs to be acknowledged. Social loneliness refers to the loneliness caused due to reduced engagement in social networks [82]. On the other hand, emotional loneliness is known to arise from the lack of an intimate and meaningful figure in a person’s life [82]. The third term, existential loneliness, has been referred to as loneliness occurring due to social disconnection caused either by voluntary pursuit of solitude or by feelings of alienation, loss of purpose in life, loss of meaningful roles, or abandonment [83]. Existential loneliness is considered to have both negative and positive dimensions depending on whether a person is seeking solitude for creative or self-exploration reasons or it is stemming from a sense of despair. One of the tools that promotes the measurement of social and emotional loneliness separately is the dJG loneliness scale. The UCLA scale measures loneliness uni-dimensionally and does not support distinctions between different types of loneliness [84]. In the context of this survey, studies [26,37] that used the dJG scale were able to report correlations between sensor-based activity measures and social and emotional loneliness scores. For example, Goonawardene et al., 2017 [26] reported that time spent out of home and daytime napping were strongly associated with social loneliness, and time spent in living room was positively correlated with emotional loneliness scores of the dJG scale. This survey also identified that sensor-based measurement of the concept of existential loneliness was indirectly explored through the existential crisis concept only in one study by Sanchez et al. [61], however, with less assessment accuracy. Otherwise, existential loneliness assessment through sensors is relatively under-explored. Given that such nuanced distinctions of different types of loneliness are under-explored, future work is needed to promote further in-depth exploration of associations between sensor-measured activity parameters and social and emotional loneliness scores derived from the dJG scale and Existential Loneliness Questionnaire [85].

### 4.10. Limitations

The survey presented in this paper focused on an overview of the activity markers, sensor technologies, study designs, co-designs, and usability aspects from existing work on sensor-based assessment of SI and loneliness in older adults. While a comprehensive search was conducted to identify work in this area, non-English articles and research articles that were not listed in the targeted databases could have been missed. This survey exclusively focused on studies conducted with older adults and did not include studies focused on younger populations. Additionally, this survey excluded articles that did not explicitly explore SI and loneliness but studied these using different conceptual descriptions of SI and loneliness.

## 5. Conclusions

This paper presented a survey on sensor-based assessment of SI and loneliness in older adults. Overall, we found that the work in this area is very limited, with most being short-term feasibility studies with a small sample size. More systematic and in-depth studies are needed in older populations, as their behavioural and lifestyle markers of SI and loneliness are likely to differ from those in younger populations and may be affected by confounding factors associated with disease or increasing frailty. Existing studies of SI assessment in older populations have briefly explored the applicability of ambient sensors. This survey identified that time spent out of home and time spent in different parts of home serve as good indicators of SI and loneliness that can be measured through ambient sensors. While privacy concerns are reported, wearable and smart phone sensors that offer ambulatory monitoring have not been explored in this population.

The suitability and acceptability of these approaches for older adults have not been explored, and hence remain open questions for further investigation. Resistance to adopt technological solutions and their under-use are some of the frequently reported concerns to consider while designing for older adults. This highlights the importance of including co-design approaches in order to incorporate consumers’ and stakeholders’ perspectives to ensure adoption and acceptance of the technology in real-world settings.

## Figures and Tables

**Table 1:** Behavioural and activity markers of social isolation and loneliness.

SI and Loneliness Activity Markers	References
Dependence for ADLs and IADLs	[32]
Physical inactivity/	[7,8,9,33]
Sedentary behaviour	[7,34]
Poor sleep quality	[7,35]
Eating and diet	[9,36]
Time out of home	[26,27,37]
Daytime dysfunction	[38]
Telephone use	[39]
Computer use	[40]

**Table 2:** Categories of activity patterns measured from sensor data.

Activity Category	Activities Measured
In-home activity	- Mobility [44], walking speed [37,44] - Time spent in different parts of home [26,27,45,46] - Activity levels in different parts of home [26,27,45]
Out-of-home activity	- Time spent out of home [26,37,44,46,47] - Frequency of going out [26,37] - Time spent in garden [27]
Sleep patterns	- Time spent in bedroom [26] - Nocturnal movement [26]
Phone usage	- Number of incoming and outgoing calls [27,44,48] - Number of incoming and outgoing text messages [27] - Duration of incoming and outgoing calls [27,48] - Persons from whom calls and texts were received/made/sent [27]

**Table 4:** Study design.

Article	Study Type	Method	Population	Sample Size	Study Duration	Co-Design
2017 [26]	Feasibility in-home trial	Mixed method	Independent living older adults	50	6 months	No
2017 [37]	Feasibility in-home trial	Quantitative	Independent-living older adults	50	6 months	No
2017 [27]	Small-scale feasibility study	Quantitative	Independent-living older adults	7	7 days	No
2016 [44]	Longitudinal feasibility study	Quantitative	Independent living older adults	16	Up to 8 months	No
2015 [61]	Small-scale feasibility study	Quantitative	Older adults	12	7 days	No
2014 [45]	Small-scale feasibility study	Quantitative	Independent living older adults	13	28 days	No
2013 [46]	Feasibility in-home trial	Quantitative	Independent living older adults	34	5 days	No

**Table 5:** Activity features extracted from sensor data.

Article	Features Extracted
Goonawardene et al., 2017 [26]	- Sleep - Going out behaviour - Toileting - Time spent in living room - Activity level in kitchen
Huynh et al., 2017 [37]	- In-home mobility patterns - Going out behaviour
Martinez et al., 2017 [27]	- Communication variables from phone - Number of incoming family calls - Average duration of incoming family calls - Number of incoming calls from friends - Average duration of outgoing calls to the family - Number of messages sent to the friends - Number of messages received from the family - Mobility variables from Bluetooth - Average time in the bedroom - Average time in the living room - Average time in the dining room - Average time in the garden - Average time in other area of the home
Austin et al., 2016 [44]	- Daily hours spent outside the home - Number of incoming and outgoing phone calls - In-home walking speed - In-home mobility - Time spent on computer - Number of sessions on computer
Lyons et al., 2015 [47]	- Time spent out of home
Petersen et al., 2015 [48]	- Number and duration of incoming and outgoing calls
Walsh et al., 2014 [45]	- Percentage of time spent in each room/location - Number of transitions between locations - Number of firings from each sensor - Total duration of activity
Petersen et al., 2013 [46]	- Time out of home - Number of sensor firings during each 5 min interval - Entry and exit events - Room from which the sensor events were recorded

**Table 6:** Types of sensors and installation locations.

Article	Types of Sensors	Installation Locations
Goonawardene et al., 2017 [26]	PIR motion sensors and door contact sensors	Living room Bedroom Kitchen Bathroom Main door
Huynh et al., 2017 [37]	Passive infra-red (PIR) sensor and reed switch.	Living room (PIR) Bedroom (PIR) Kitchen (PIR) Bathroom (PIR) Main door (Reed)
Martinez et al., 2017 [27]	Bluetooth devices and a mobile phone application	Bedroom Living room Dining room Garden Other areas such as the studio, garage, kitchen, and cellar
Austin et al., 2016 [44]	Wireless pyroelectric infrared motion sensors	Each room of the home
	Magnetic contact sensors	Outside of the home doors
	Phone monitors (Shenzhen Fiho Electronic, Fi3001B) plug	Plugged into the phone
	Computer monitoring software programs	Installed on the computer
Sanchez et al., 2015 [61]	GPS sensor	Smartphone
Walsh et al., 2014 [45]	PIR sensors	Living room Hall Bedroom
	Light switch sensor	Water closet Living room Kitchen Main bedroom 2nd bedroom en suite
	Door contact sensor	Front door Rear door Living room Main bedroom door En Suite door
Petersen et al., 2013 [46]	Pyroelectric motion sensors (MS16A, x10.com)	In each room
	Contact sensors (DA10A, x10.com)	On refrigerator Outside doors of home
	Motion-activated video cameras (Logitech C600)	Over the home door

**Table 7:** Summary of data analysis methods and key findings.

Article	Measured Parameter	Scales Used	Data Analysis Method	Findings	
				Activity/Feature	Association with Loneliness/SI Score
Goonawardene et al. [26]	SI	Lubben Social Network Scale, dJG, Custom Survey, GDS, Abbreviated Mental Test Score, PQSI, Katz ADL Scale, Lawton IADL Scale	Correlation analysis	Time out of home	Associated with social loneliness score but not with Emotional Loneliness Score Significantly negatively correlated with SI
				Time spent in living room	Significantly correlated with emotional loneliness score
				Daytime napping	Significant positive correlation with social loneliness
				GDS	Significant positive correlation with composite SI score
				Lawton and Brody IADL and PSQI sleep quality	Significant correlation with emotional loneliness score
Huynh et al. [37]	Loneliness	dJG Scale, GDS	Correlation analysis	Frequency of going out of home	Highest correlation with loneliness
				Time spent in kitchen	Significant correlation with loneliness level
Martinez et al. [27]	SI	Lubben Social Network Scale,	Predictive modelling	N/A	N/A
Austin et al. [44]	Loneliness	University of California Los Angeles (UCLA) Loneliness scale	Longitudinal linear mixed effects regression modelling	Time out of home	Negatively associated with loneliness
				Number of computer sessions	Significantly correlated with loneliness score
Sanchez et al. [61]	Loneliness	ESTE-R	Predictive modelling	N/A	N/A
Walsh et al. [45]	Loneliness	De Jong Gierveld Scale, CES-D, HADS, PSQI, MOCA, SF-36, IADL	Correlation analysis and principle component analysis	Time spent in living room	Associated with increased loneliness, anxiety, and poor sleep and inversely with IADL
				Time spent in bed room	Inversely associated with anxiety, depression, IADL, and poor sleep
				Sleep/nocturnal movement	Inversely correlated with IADL and positively associated with loneliness score
				Time out of home	associated with IADL
Petersen et al. [46]	Loneliness	UCLA Loneliness Scale, Berkman’s Social Disengagement Index	Logistic regression-based classifier and correlation analysis	Time out of home	Inversely correlated with loneliness score

## Data Availability

Not applicable.

## Abbreviations

The following abbreviations are used in this manuscript:

dJG	De Jong Gierveld
GDS	Geriatric Depression Scale
ML	Machine Learning
PIR	Passive Infra-red
SI	Social Isolation
UCLA	The University of California Los Angeles
